# Assessment of Knowledge on Cardiovascular Disease Risk Factors Among Diabetes Mellitus Patients in Tikur Anbessa Specialized Hospital, Ethiopia 2021: A Cross-Sectional Study

**DOI:** 10.1155/nrp/7737392

**Published:** 2025-04-19

**Authors:** Filipos Alemu, Lemlem Beza, Tilahun Jiru, Dereje Endale

**Affiliations:** ^1^Department of Nursing, College of Health Science, Dilla University, Dilla, Ethiopia; ^2^Department of Emergency Medicine, College of Health Science, Addis Ababa University, Addis Ababa, Ethiopia

**Keywords:** cardiovascular disease, diabetes mellitus, Ethiopia, knowledge, risk factors

## Abstract

**Introduction:** Cardiovascular diseases (CVDs) are a growing problem with increasing global prevalence and the most common cause of mortality worldwide. Knowledge about the disease and risk factors reduces exposure to modifiable risk factors and, as a result, contributes to prevention. As diabetes is a prevalent disease and there is limited research about CVD risk factors in Ethiopia, we conducted a study to assess this knowledge.

**Methods:** A cross-sectional study was conducted on diabetes mellitus patients on follow-up at Tikur Anbessa Specialized Hospital from April 11 to May 16, 2021. The participants were selected using a consecutive sampling method. Knowledge was measured using a heart disease fact questionnaire, and a score of less than 70% was defined as suboptimal. Data were analyzed using SPSS Version 26.0. Associations between dependent and independent variables were identified based on AOR, with 95% CI and a *p* value less than or equal to 0.05.

**Result:** A total of 404 patients with a mean age of 52.03 ± 14.39 participated in the study, and more than half, 217 (53.7%), of patients were females. About half of the patients (52%) had good knowledge of CVD risk factors. In multivariable logistic regression, urban residency (AOR, 3.335; 95% CI [1.134–9.809]), higher educational level (AOR, 4.016; 95% CI [1.78–9.061]), being employed (AOR, 1.942; 95% CI [1.058–3.566]), and hearing information about CVD risk factors (AOR, 2.492; 95% CI [1.573–3.949]) are associated with knowledge of CVD risk factors.

**Conclusion:** This study revealed that almost half of diabetes mellitus patients had suboptimal knowledge about CVD risk factors. Urban residence, higher education level, employment, and information about CVD risk factors are positively associated with good knowledge of CVD risk factors. Health education is needed to improve their knowledge.

## 1. Introduction

Cardiovascular disease (CVD) refers to conditions that affect the heart and blood vessels [1, 2]. Risk factors for CVD are any habits or behaviors that increase an individual's risk of developing CVD and may be modifiable or nonmodifiable [2–4]. The prevalence of CVDs is rising globally and is one of the leading causes of mortality worldwide [5–8]. Among deaths, attributed to noncommunicable diseases (NCDs), nearly half of the deaths are a result of CVD, with an estimated annual 17.3 million deaths, and 10% of the global disease disability-adjusted life year (DALY) burden. From these deaths, over three-quarters of CVD deaths occur in low- and middle-income countries. In developing nations, the burden of CVD is significant, yet the knowledge of the disease and its associated risk factors remains limited. Moreover, findings show that the prevalence of CVD is increasing and posing a public health challenge in these regions [9, 10].

Diabetes mellitus (DM) is the main risk factor for CVD [11, 12] because over time, high blood glucose levels can damage blood vessels and nerves that innervate the heart [13]. Diabetic heart disease arises from different mechanisms contributing together, some of them are: oxidative stress, inflammation, alteration in substrate metabolism, and myocardial death [14]. The global number of individuals with diabetes in 2019 was estimated to be 463 million (9.3% of the world's population), a figure projected to rise to 578 million (10.2%) by 2030, and to 700 million (10.9%) by 2045 [15]. Atherosclerotic cardiovascular disease (ASCVD) is the leading cause of morbidity and mortality among patients with DM. It is the greatest reason for the direct and indirect costs of DM [5]. Individuals with diabetes are about three times more prone to have CVD than those without diabetes, and death in people with diabetes is most commonly due to cardiovascular complications [12, 16, 17]. DM not only increases the chance of developing CVD but also adversely affects the outcome; around 70% of deaths among diabetes patients are due to CVD [18–21].

The rise in cardiovascular problems around the globe is due to significant worldwide changes in behavior and lifestyle, such as poor diet, tobacco use, physical inactivity, and excess alcohol consumption, which mainly result from inadequate awareness of their effect on cardiovascular health [22]. Evidence from studies in both developed and developing countries indicates that knowledge of CVD risk factors is inadequate [11, 23–25], which is a great tackle in combating this escalating problem [26–28]. In most studies, having a diploma or higher education and residing in urban areas are associated with better knowledge [29, 30].

Ethiopia is among the first four countries in sub-Saharan Africa with the highest prevalence of DM and related hospital admissions. According to the 2017 estimate by the International Diabetes Federation (IDF), there are 2.57 million (5.2%) adults aged 20–79 years living in Ethiopia [31]. To the best of my knowledge, no published study examined the knowledge of DM patients regarding CVD risk factors in Ethiopia. Understanding CVD risk factors is crucial for individuals to actively decrease their risk, since many of the risk factors are modifiable [23, 32]. Patients' perceptions of their CVD risk can significantly influence their decision-making regarding self-management and behavior [11]. Knowledge of heart disease risk factors is essential for making an informed decision that can reduce an individual's overall cardiovascular risk. This study assessed knowledge of CVD risk factors and associated factors among DM patients.

## 2. Methods

### 2.1. Study Design

A cross-sectional study was conducted to assess knowledge of risk factors of CVD and associated factors among diabetes patients.

### 2.2. Study Setting and Period

The study was conducted at a DM follow-up clinic in Tikur Anbessa Specialized Hospital, Addis Ababa, Ethiopia (TASH). It contains over 700 beds and is the largest teaching hospital in the country, providing healthcare services to more than 700,000 patients annually. The diabetes clinic is one of the various specialty clinics hosted by TASH, and it serves diabetes patients referred from all corners of the country. The hospital is located in Addis Ababa, the capital city of Ethiopia and the seat of the African Union [33].

*Study period*: The study was conducted from April 11 to May 16, 2021.

### 2.3. Study Participants

The source population is all DM patients visiting TASH. Patients aged 18 and above who attended the TASH DM clinic for follow-up during the study period were included in the study. Diabetic patients with hearing and talking impairments, as well as those who were critically ill, were excluded, because these issues would hinder their ability to participate in the study.

### 2.4. Sample Size Determination and Sampling

The sample size was calculated using the single population proportion formula, assuming a 95% confidence interval, a 5% margin of error, and a 50% proportion of DM patients with good knowledge of CVD risk factors. Based on these assumptions, the sample size was determined to be 384, and by adding a 10% nonresponse rate, the final sample size was adjusted to 423. Consecutive sampling was employed to recruit study participants. DM patients who fulfilled the inclusion and exclusion criteria were included until the final sample size was achieved.

### 2.5. Variables

#### 2.5.1. Dependent Variable

In this study, the dependent variable is the knowledge of DM patients regarding CVD risk factors.

#### 2.5.2. Independent Variables

Age, sex, marital status, educational level, residency, family history of CVD, having CVD, duration of treatment for DM, hearing information about CVD risk factors, occupation, and income level are independent variables.

### 2.6. Data Collection Technique and Tools

Face-to-face interviews with patients were conducted by four BSc nurses by using structured questionnaires to collect information from each participant. Data were collected using two tools: one for socio-demographics and the other for knowledge of CVD risk factors, comprising a total of 35 questions. The Heart Disease Fact Questionnaire (HDFQ), adapted from a previous study, was used to assess knowledge of CVD risk factors [34]. Developed by Wanger et al., the HDFQ demonstrated good content and face validity along with adequate internal consistency as indicated by a Kuder-Richardson-20 formula score of 0.77 [35]. In the current study, the reliability of HDFQ, assessed by Cronbach's alpha, showed a good internal consistency of 0.83.

### 2.7. Data Quality Assurance

The questionnaire was pretested before data collection, and necessary corrections were made. In addition to this, 2-day training was given to data collectors and supervisors about the study and the content of the questionnaire in detail. Furthermore, continuous and close supervision of the data collection process, along with proper categorization and coding of the data, was ensured. The questionnaire was translated into Amharic and back-translated to English to verify the reliability of the translation. The principal investigators and supervisor checked the completeness and consistency of data daily.

### 2.8. Data Entry and Analysis

After data collection, it was checked for completeness and consistency. Coded data were entered into an EpiData entry version 4.6. After that, it was exported and analyzed by using SPSS Version 26. Descriptive statistics such as percentages, frequencies, means, and standard deviation were used to summarize the patient's characteristics. The patient's knowledge of the CVD risk factor was measured on a two-point scale with “0” = the wrong answer and “1” = the correct answer. The total score was identified by adding the correct score of all items.

Bivariable logistic regression analysis was conducted, and variables with a *p*-value ≤ 0.25 in this model were included in the multivariable logistic regression model. Adjusted odds ratio with a 95% confidence interval was used to identify the significant predictors of good knowledge with a *p* value cutoff of ≤ 0.05. Before executing the final logistic regression model, we checked for multicollinearity, and the model was deemed fit, with the Hosmer and Lemeshow test being insignificant (*p* > 0.05).

### 2.9. Operational Definition

*Having CVD*: This means the participants have confirmed heart disease, stroke, and blood vessel disease.

*Duration of treatment for DM*: This refers to the number of years they have been receiving treatment for DM.

*Hearing information about CVD risk factors*: This means getting any type of information regarding risk factors for CVD.

*Good knowledge of CVD risk factors*: It is defined as scoring ≥ 70% in HDFQ [9, 10, 34].

*Poor knowledge of CVD risk factors*: It is defined as scoring < 70% in HDFQ [9, 10, 34].

### 2.10. Ethical Consideration

Ethical approval was obtained from the emergency medicine department ethical review committee, by letter with “Ref No: EM/SM/2016/2021” before the commencement of the study. Written informed consent was obtained from all the study participants during the data collection process. The information gathered from the participants was kept confidential.

## 3. Result

### 3.1. General Characteristics of the Study Participants

A total of 404 participants were included in the study, resulting in a response rate of 95.5%. The mean age of respondents was 52.03 ± 14.39, with the majority being 217 (53.7%) females. Regarding their marital status, more than four-fifths of participants were ever married, 340 (84.2%) (Table 1).

### 3.2. Information About CVD Risk Factors

In this study, 196 (48.5%) respondents received information about CVD risk factors. Among those informed about CVD risk factors, 166 (41.1%) of respondents received the information from healthcare workers, 88 (21.8%) from media (radio, television, and reading), and 29 (7.2%) from friends and relatives (Table 2).

### 3.3. Item-Wise Response to HDFQ

The most common CVD risk factor identified by the participants was that “regular physical activity will lower a person's chance of getting heart disease” 366 (90.6%), followed by smoking, being overweight, DM, and high blood pressure, 359 (88.9%), 356 (88.1%), 343 (84.9%), and 336 (83.2%), respectively. Among the participants, more than half, 53.5% (216), were unaware that a family history of CVD is a risk factor. Almost one-fifth 63 (18.9%) were not aware that keeping blood pressure under control decreases the risk of developing CVD, 100 (24.8%) were unable to recognize “eating fatty food affects blood cholesterol levels,” and 61 (15.1%) did not consider DM as a risk factor for CVD (Table 3).

### 3.4. Knowledge Score of CVD Risk Factors

**Figure 1:**
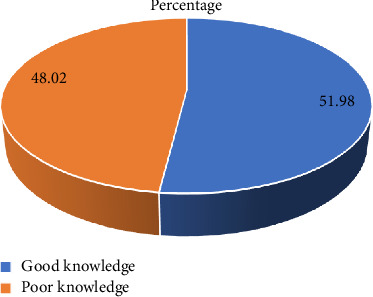
Level of knowledge of CVD risk factors of the participants.

The level of knowledge regarding CVD risk factors among DM patients was assessed through their correct responses to 22 knowledge questions. The participants scored a minimum of two and a maximum of 22, with a mean score of 14.75 ± 4.18. The median and mode of the knowledge score were 16 and 18, respectively. Their knowledge was categorized into good and poor using a 15.4 (70%) knowledge score as a cut-off point. In terms of overall knowledge of CVD risk factors, approximately half of the participants 194 (48.0%) had poor knowledge, giving correct responses to < 16, HDFQ, while 210 (52%) of the participants demonstrated good knowledge by giving correct responses to ≥ 16, HDFQ (Figure 1).

### 3.5. Factors Associated With CVD Risk Factors

Bivariable logistic regression was performed to select variables for the final model using *p* ≤ 0.25. Residence, sex, age, income, CVD history in the family, information about CVD risk factors, educational level, occupation, and marital status were included in the final model. In multivariable logistic regression, place of residency, education level, occupation, and information about CVD risk factors remained significantly associated with knowledge of CVD risk factors.

This study observed that urban residents are three times more likely to have good knowledge compared to rural residents AOR = 3.335, 95% CI (1.134–9.809), *p*-value = 0.040. Similarly, those who attended high school or less are two times more likely to have good knowledge than those who do not have formal education, AOR = 2.374, 95% CI (1.192–4.726). When we compare participants with diplomas and above to those who have not attended formal education, the former are four times more likely to have good knowledge, AOR = 4.016, 95% CI (1.780–9.061). Employed participants are more likely to have good knowledge compared to those not employed, AOR = 1.942, 95% CI (1.058–3.566). Finally, participants who received information about CVD risk factors are twice as likely to have good knowledge as those who did not, AOR = 2.492, 95% CI (1.573–3.949) (Table 4).

## 4. Discussion

In this institution-based study, we found that most participants were female, over 80% had attended high school or higher, and approximately 44.1% were employed. Participants' knowledge of CVD risk factors was suboptimal, with roughly half of the participants having an overall poor knowledge score. Educational level, occupation, residency, and hearing information about CVD risk factors were predictors of good knowledge.

Most of the study participants identified smoking 359 (88.9%), being overweight 356 (88.1%), and high blood pressure 336 (83.2%) as risk factors for CVD. This study finding is relatively consistent with a study conducted in northern Ethiopia, where smoking 280 (97.6%), being overweight 262 (91.3%), and having high blood pressure were identified as risk factors for CVD 235 (81.8%) [34]. Similarly, in the study done in Kuwait, more than 80% of participants identified smoking, being overweight, and having high blood pressure as risk factors for CVD. [32]. In contrast, a study conducted in Cameroon shows a significant difference, in which about 69.7% of overweight, 67.0% lack of exercise, and 73.3% high blood pressure are mentioned as risk factors for CVD [36]. This disparity could be due to differences in sociocultural characteristics of the participants.

Knowledge of CVD risk factors among DM patients was not satisfactory, with about half of the participants having suboptimal knowledge, with a mean score of 67.01%. This finding is similar to previous study results reported from India and South Africa [37, 38]. Contrary to this finding study done in Zimbabwe about knowledge of CVD risk factors, the mean score was 56.25%, which is lower than the current study finding [25]. The discrepancy may be due to sample size differences; in the earlier study, only 67 participants were involved.

In the present study, education level was associated with good knowledge of CVD risk factors. Consistent with this finding, numerous studies [23, 32, 36, 39] have shown that higher education is associated with good knowledge of CVD risk factors. This may be attributed to the fact that as educational level increases, health information–seeking behavior also increases, and they can easily apprehend information about CVD risk factors, which aids them in making informed judgments and decisions regarding their health in everyday life [40].

A systematic review done in sub-Saharan Africa indicates a significant association between residence and knowledge of CVD risk factors, with urban residents having better knowledge than their rural counterparts [29]. This finding aligns with the current study, which shows that urban residents are three times more likely to have good knowledge compared to those in rural areas. Consequently, the higher knowledge of CVD risk factors in urban residents could be due to their better access to health information compared to rural residents, and attainment of a higher educational level is high among urban residents [41], which could enhance individuals' information-seeking behavior.

Additionally, the current study shows that employed individuals have higher levels of knowledge regarding CVD risk factors compared to those who are not employed. Similarly, a study done in Uganda reveals that formally employed participants are more knowledgeable than their counterparts [42]. Employment enhances the chance of getting more health-related information, and better knowledge about CVD risk factors may result from higher health literacy among employed ones [43]. Furthermore, a current study demonstrates that participants who received information about CVD risk factors are twice as likely to have good knowledge compared to those who have not, a finding similar to research by Ndejjo et al. [42]. It is evident that having information on CVD risk factors enhances knowledge and influences behavior, ultimately improving the well-being of an individual [44, 45].

Our study mirrors that economic and social factors are determinants of health knowledge and behavior change [46], highlighting the importance of considering these factors during health education provision. It is essential to develop and integrate tailored discussion programs into routine nursing DM care to address this issue effectively. Moreover, further investigating the effectiveness of educational interventions in enhancing knowledge about CVD risk factors, as well as understanding how socio-demographic factors influence this knowledge, is important. Low health knowledge is associated with adverse health outcomes and poor use of healthcare services [30]. Poor knowledge of CVD risk factors hurts attitude, practice, compliance with treatment, and health behavior. Improving CVD risk factors knowledge is necessary for better health outcomes.

### 4.1. Limitations

This study may be subject to bias, because the subject may be prone to social desirability bias, and an effect relationship cannot be established because the design is cross-sectional. The study was done in a single setting, and a consecutive sampling method was used; this may influence the generalizability of the findings. Some sociodemographic variables, such as religion, ethnicity, and accessibility of health facilities, were not included in the study.

## 5. Conclusion

Overall, this study was done on DM patients in follow-up in TASH and shows that DM patients had unsatisfactory knowledge about CVD risk factors, with almost half of the patients having suboptimal knowledge. Also, health education about CVD risk factors was not adequate. Higher education, being employed, being an urban resident, and having information about CVD risk factors are associated with good knowledge. Our study suggests that measures should be implemented to improve the knowledge of DM patients about CVD risk factors. So, appropriate health education on the CVD risk factors should be provided.

## Figures and Tables

**Table 1:** General characteristics of the study participants.

Variables		Frequency	Knowledge	
			Poor	Good
Sex	Male	187 (46.3%)	75	112
	Female	217 (53.7%)	119	98

Age	18–40	97 (24.0%)	50	47
	41–60	191 (47.3%)	84	107
	≥ 61	116 (28.7%)	60	56

Residence	Urban	376 (93.1%)	171	205
	Rural	28 (6.9%)	23	5

Marital status	Never married	64 (15.8%)	35	29
	Ever married	340 (84.2%)	159	181

Duration of treatment for diabetes mellitus	Less than 6 years	144 (35.6%)	74	70
	Six years and above	260 (64.4%)	120	140

Level of education	No formal schooling	80 (19.8%)	63	17
	High school and less	186 (46.0%)	93	93
	Diploma and above	138 (34.2%)	38	100

Occupation	Employed	178 (44.1%)	64	114
	Retired	79 (19.6%)	28	51
	Not employed	147 (36.4%)	102	45

Family history of CVD	Yes	61 (15.1%)	23	38
	No	343 (84.9%)	171	172

Having CVD	Yes	179 (55.7%)	88	91
	No	225 (44.3%)	106	119

Monthly income	< 5000	343 (84.9%)	181	162
	5001–10,000	51 (12.6%)	11	40
	> 10,000	10 (2.5%)	2	8

Type of diabetes mellitus	Type I	108 (26.7%)	55	53
	Type II	294 (73.3%)	139	157

**Table 2:** Study participants who received information about CVD risk factors and their source of information.

Variable		Frequency	Knowledge	
			Poor	Good
Did you get information about the risk factors of CVD?	Yes	196 (48.5%)	65	131
	No	208 (51.5%)	129	79

**Source of information**		**Frequency**	**Percentage (%)**	

Healthcare providers		166	41.1	
Friends and relatives		29	7.2	
Media		88	21.8	

*Note:* For the source of information, frequencies cannot be added to the given *N*, and the sum of percentages cannot be 100% due to multiple responses.

**Table 3:** Responses of the participants to HDFQ.

Questions (*n* = 404)	Correct response	Frequency
People can easily know by themselves when they have heart disease	False	107 (26.5%)
If you have a family history of heart disease, you are at risk for developing heart disease	True	188 (46.5%)
The older a person is, the greater their risk of having heart disease	True	253 (62.6%)
Smoking is a risk factor for heart disease	True	359 (88.9%)
A person who stops smoking will lower their risk of developing heart disease	True	360 (89.1%)
High blood pressure is a risk factor for heart disease	True	336 (83.2%)
Keeping blood pressure under control will reduce a person's risk of developing heart disease	True	331 (81.9%)
High cholesterol is a risk factor for developing heart disease	True	307 (76.0%)
Eating fatty foods does not affect blood cholesterol levels	False	304 (75.2%)
If your “good” cholesterol (HDL) is high, you are at risk for heart disease	False	67 (16.6%)
If your “bad” cholesterol (LDL) is high, you are at risk for heart disease	True	112 (27.7%)
Being overweight increases a person's risk for heart disease	True	356 (88.1%)
Regular physical activity will lower a person's chance of getting heart disease	True	366 (90.6%)
Only exercising at a gym in an exercise class will help lower a person's chance of developing heart disease	False	289 (71.5%)
Walking and gardening are considered exercises that will help lower a person's chance of developing heart disease	True	299 (74.0%)
Diabetes is a risk factor for developing heart disease	True	343 (84.9%)
A person who has diabetes can reduce their risk of developing heart disease if they keep their blood sugar level under control	True	339 (83.9%)
A person who has diabetes can reduce their risk of developing heart disease if they keep their blood pressure under control	True	335 (82.9%)
A person who has diabetes can reduce their risk of developing heart disease if they keep their weight under control	True	328 (81.2%)
People with diabetes rarely have high cholesterol	False	165 (40.8)
People with diabetes tend to have low HDL “good” cholesterol	True	81 (20.0%)
If your blood sugar is high over several months, it can cause your cholesterol level to go up and increase your risk of heart disease	True	333 (82.4%)

**Table 4:** Factors associated with good knowledge of CVD risk factors.

Variables	Knowledge of risk factors		COR (95% CI)	AOR (95% CI)	*p* value
	Poor	Good			
Sex					
Male	75	112	1.813 (1.22–2.695)	1.076 (0.664–1.743)	0.767
Female	119	98	1	1	
Age group					
18–40	50	47	1	1	
41–60	84	107	1.355 (0.83–2.212)	0.721 (0.381–1.363)	0.314
61 and above	60	56	0.993 (0.579–1.703)	0.643 (0.290–1.427)	0.278
Residence					
Urban	171	205	5.515 (2.053–14.814)	3.335 (1.134–9.809)	0.029^∗^
Rural	23	5	1	1	
Marital status					
Never married	35	29	1	1	
Ever married	159	181	1.374 (0.804–2.349)	1.917 (0.946–3.884)	0.071
Level of education					
No formal schooling	63	17	1	1	
High school and less	93	93	3.706 (2.018–6.806)	2.374 (1.192–4.726)	0.014^∗^
Diploma and above	38	100	9.752 (5.076–18.738)	4.016 (1.780–9.061)	0.001^∗^
Occupation					
Employed	64	114	4.037 (2.535–6.431)	1.942 (1.058–3.566)	0.032^∗^
Retired	28	51	4.129 (2.313–7.368)	1.909 (0.889–4.097)	0.097
Not employed	102	45	1	1	
Family history of CVD					
Yes	23	38	1.643 (0.939–2.874)	1.112 (0.588–2.102)	0.743
No	171	172	1	1	
Information about the risk factors of CVD					
Yes	65	131	3.291 (2.187–4.951)	2.492 (1.573–3.949)	0.000^∗^
No	129	79	1	1	
Monthly income					
< 5000	181	162	1	1	
5001–10,000	11	40	4.063 (2.017–8.183)	1.340 (0.586–3.061)	0.488
> 10,000	2	8	4.469 (0.935–21.351)	1.927 (0.368–10.087)	0.437

Abbreviations: AOR, adjusted odds ratio; COR, crude odds ratio.

^∗^Statistically significant at *p* ≤ 0.05 in AOR.

## Data Availability

The data of this study can be accessed from the corresponding author upon reasonable request.

## Nomenclature

ASCVD: Atherosclerotic cardiovascular disease
CVD: Cardiovascular disease
DALYS: Disability adjusted life years
DM: Diabetes mellitus
HDFQ: Heart Disease Fact Questionnaire
HF: Heart failure
IDF: International Diabetes Federation
NCD: Noncommunicable disease
